# Halved contrast medium dose in lower limb dual-energy computed tomography angiography—a randomized controlled trial

**DOI:** 10.1007/s00330-023-09575-3

**Published:** 2023-04-18

**Authors:** Cathrine Helgestad Kristiansen, Owen Thomas, Thien Trung Tran, Sumit Roy, Dan Levi Hykkerud, Audun Sanderud, Jonn Terje Geitung, Peter M. Lauritzen

**Affiliations:** 1 Health Faculty, Oslo Metropolitan University, Oslo, Norway; 2 Department of Diagnostic Imaging and Intervention, Akershus University Hospital, Lørenskog, Norway; 3 Health Services Research Department (HØKH), Akershus University Hospital, Lørenskog, Norway; 4 Institute of Clinical Medicine, University of Oslo, Oslo, Norway; 5 Division of Radiology and Nuclear Medicine, Oslo University Hospital, Oslo, Norway

**Keywords:** Computed tomography angiography, Contrast media, Peripheral artery disease

## Abstract

**Objectives:**

To compare vascular attenuation (VA) of an experimental half iodine-load dual-layer spectral detector CT (SDCT) lower limb computed tomography angiography (CTA) with control (standard iodine-load conventional 120-kilovolt peak (kVp) CTA).

**Methods:**

Ethical approval and consent were obtained. In this parallel RCT, CTA examinations were randomized into experimental or control. Patients received 0.7 vs 1.4 mL/kg of iohexol 350 mgI/mL in the experimental- vs the control group. Two experimental virtual monoenergetic image (VMI) series at 40 and 50 kiloelectron volts (keV) were reconstructed. Primary outcome: VA. Secondary outcomes: image noise (noise), contrast- and signal-to-noise ratio (CNR and SNR), and subjective examination quality (SEQ).

**Results:**

A total of 106 vs 109 were randomized and 103 vs 108 were analyzed in the experimental vs, control groups, respectively. VA was higher on experimental 40 keV VMI than on control (*p* < 0.0001), but lower on 50 keV VMI (*p* < 0.022). Noise was higher on experimental 40 keV VMI than on control (*p* = 0.00022), but lower on 50 keV VMI (*p* = 0.0033). CNR and SNR were higher than the control on experimental 40 keV VMI (both *p* < 0.0001) and 50 keV (*p* = 0.0058 and* p* = 0.0023, respectively)**.** SEQ was better on both VMIs in the experimental group than in the control (both *p* < 0.0001).

**Conclusions:**

Half iodine-load SDCT lower limb CTA at 40 keV achieved higher VA than the control. CNR, SNR, noise, and SEQ were higher at 40 keV, while 50 keV showed lower noise.

**Clinical relevance statement:**

Spectral detector CT with low-energy virtual monoenergetic imaging performed halved iodine contrast medium (CM) lower limb CT-angiography with sustained objective and subjective quality. This facilitates CM reduction, improvement of low CM-dosage examinations, and examination of patients with more severe kidney impairment.

**Trial registration:**

Retrospectively registered 5 August 2022 at clinicaltrials.gov NCT05488899.

**Key Points:**

• *Contrast medium dosage may be halved in lower limb dual-energy CT angiography with virtual monoenergetic images at 40 keV, which may reduce contrast medium consumption in the face of a global shortage.*

• *Experimental half-iodine-load dual-energy CT angiography at 40 keV showed higher vascular attenuation, contrast-to-noise ratio, signal-to-noise ratio, and subjective examination quality than standard iodine-load conventional.*

• *Half-iodine dual-energy CT angiography protocols may allow us to reduce the risk of PC-AKI, examine patients with more severe kidney impairment, and provide higher quality examinations or salvage poor examinations when impaired kidney function limits the CM dose.*

## Introduction

Peripheral artery disease (PAD) is an important cause of death and disability worldwide [1]. PAD is characterized by atherosclerotic stenosis or occlusion in peripheral arteries, usually in the lower limbs, and the prevalence increases significantly with age [1].

Computed tomography angiography (CTA) is considered the examination of choice in PAD due to its availability, non-invasive nature, fast image acquisition, cost efficiency, and high diagnostic accuracy [2–4]. Although the incidence is low, post-contrast acute kidney injury (PC-AKI) remains a risk with CTA, especially for patients with severely impaired kidney function [5–7]. Reducing the contrast medium (CM) dosage may lessen this risk, which is worthwhile since PAD is associated with impaired kidney function, and lower limb CTA usually requires a large CM volume [8–11].

Reduction of CM dosage in CT, based on the increasing attenuation of iodine as photon energies approach the k-edge at 33.2 keV, may be accomplished with low kilovolt peak (kVp) or dual energy monoenergetic reconstruction [12].

Since dual-energy CT (DECT) was first described in 1973, vendors have introduced different techniques [13]. Dual-layer spectral detector CT (SDCT) uses one x-ray tube and a two-layer detector with different sensitivity for the low- and high photon spectrum energies. SDCT always captures spectral information at 120/140 kVp, so imaging protocols need not be modified for dual-energy scanning. The conventional images from the two detector layers are of equal quality to conventional scanners [14, 15].

With DECT, virtual monoenergetic images (VMI) are widely used to increase iodine attenuation and image contrast, especially in vascular imaging. The lowest energy levels of 40 and 50 keV, roughly equivalent to 70 and 80 kVp on single energy scanners, are the most promising with potential reductions in CM of 60%and 40%, respectively [16, 17]. VMI may also improve examination quality or salvage examinations with poor enhancement [18]. Few studies have reported on VMI in lower limb CTA.

Imaging protocols with greatly reduced CM-dosages may only be applied if clinical feasibility is ascertained, and the examination quality is adequate, and preferably equal or improved compared to conventional imaging [15].

Our hypothesis was that we could achieve a higher vascular attenuation by a half iodine-load SDCT lower limb CTA at 40 or 50 keV than with standard iodine-load at 120 kVp, and our primary objective was to compare the VA of these acquisitions. Our secondary objectives were to assess and compare contrast-to-noise ratio (CNR), signal-to-noise ratio (SNR), image noise (noise), and subjective examination quality (SEQ) between the two groups.

## Methods

### Patient population

Between 28 January 2019 and 16 October 2020, patients referred for lower limb CTA at University Hospital were considered for enrolment in this single-centre, parallel, randomized controlled trial (RCT). The inclusion criteria were: estimated glomerular filtration rate (eGFR) > 30 mL/min/1.73 m^2^ and clinical suspicion of- or known lower limb PAD. The exclusion criteria were: contraindication to iodinated CM, age < 18 years, pregnancy, or critical ischemia.

The study was approved by the Regional Committee for Medical and Health Research Ethics of South East Norway (ref 2018/473) and the Data Protection Officer of the hospital. All participants gave written informed consent. The study was retrospectively registered on 5 August 2022 at ClinicalTrials.gov (NCT05488899).

### Randomisation

Examinations were randomized to either the experimental or control group. The principal investigator performed randomisation with a 1:1 ratio using the RANDBETWEEN (1,2) function in Microsoft Excel (Microsoft Corporation) and sealed the randomisation codes in sequentially numbered envelopes. Enrolment and allocation were performed by radiographers in the CT lab. The patients and personnel assessing outcomes were blinded to the randomisation.

### Imaging protocol

We used a bodyweight-adapted CM volume of iohexol 350 mgI/mL (Omnipaque 350, GE Healthcare). The control group received the standard iodine load of 1.4 mL/kg, while the experimental group received 0.7 mL/kg as the CM was diluted 1:1 with saline. This allowed identical injection rate and -time in both groups.

The CM was administered using a power injector (CT Exprès TM 4D, Bracco Injeneering SA), with a 50 mL saline flush. We used an automatic bolus tracker with a region of interest (ROI) in the abdominal aorta (AA), 120 Hounsfield units (HU) trigger point, and scan after 15 s. The maximum injected volume was limited to 130 mL, and the minimum to 60 mL.

Scans were performed supine, feet-first on a Philips IQon SDCT scanner (Philips Healthcare). The scan parameters were: tube voltage: 120 kVp, collimation: 64*0.625 mm, rotation time: 0.5 s, pitch: 1.171, matrix: 512*512, slice thickness: 0.9 mm, increment: 0.45 mm. Automatic tube current (DoseRight 3D-DOM, Philips Healthcare) was enabled, and the Dose Right Index was set at 21.

Conventional images were reconstructed with hybrid iterative reconstruction, iDose 4, level 3, and VMI in 40 and 50 keV with a spectral reconstruction algorithm: spectral B, denoising level 3 on an IntelliSpace Portal 9.0 workstation (Philips Healthcare). In the control group, measurements from the conventional images were analyzed. In the experimental group, measurements from the VMIs at 40 and 50 keV were analyzed. All images were assessed in the axial plane with a 1 mm slice thickness.

### Objective examination quality

One radiographer performed the analysis of objective examination quality (OEQ).

ROIs were placed manually in the AA at the mid-point between the renal arteries and the aortic bifurcation and bilaterally in.

- the common iliac arteries (CIA) at the mid-point between the aortic bifurcation and internal iliac artery,
- the superficial femoral arteries (SFA) 10 cm below the branching of the deep femoral artery, and
- the P3 segment of the popliteal artery (PA).

Each ROI was drawn as large as possible to assess as much of the arterial lumen as possible without including the arterial wall or plaques. ROIs were copied and pasted in the VMI series to ensure measurements from identical areas. We recorded VA and standard deviation (SD). Background attenuation was measured in the closest muscle. OEQ was defined as VA (primary outcome), noise, CNR, and SNR (secondary outcomes). Noise was defined as SD of VA

$$\mathrm{CNR}=\left({\mathrm{Attenuation}}_{\mathrm{artery}}-{\mathrm{Attenuation}}_{\mathrm{muscle}}\right)/{\mathrm{Noise}}_{\mathrm{artery}}\;\mathrm{and}\;\mathrm{SNR}={\mathrm{Attenuation}}_{\mathrm{artery}}/{\mathrm{Noise}}_{\mathrm{artery}}\;\mathrm{were}\;\mathrm{calculated}.$$

### Subjective examination quality

SEQ (secondary outcome) was independently rated by two vascular interventional radiologists. SEQ was rated in the AA, CIA, SFA, PA, and calf arteries. In the case of the three calf arteries, the artery with the best demarcation of the lumen was rated. In cases of occlusion, SEQ was not assessed at that anatomical level.

**Fig. 1:**
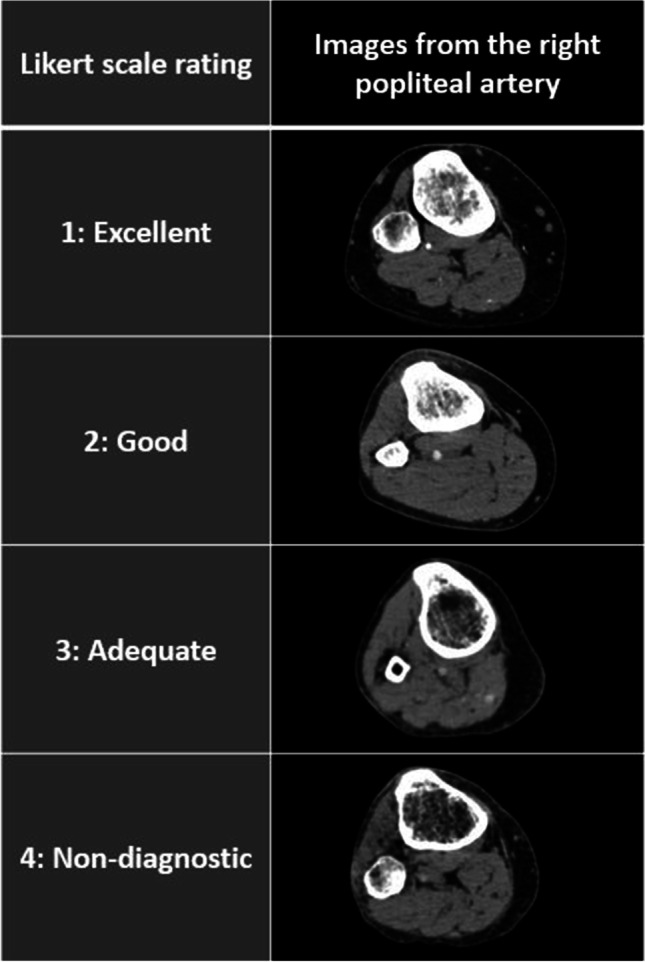
Likert scale for subjective examination quality

A 4-point rating scale was used, as follows: 1: Excellent, 2: good, 3: adequate, 4: non-diagnostic, where ratings 1 through 3 were considered diagnostic quality (Fig. 1). If both radiologists rated SEQ as diagnostic quality, the mean of the two scores was used for statistical analysis. Scores of 1.5 and 2.5 were termed “very good” and “quite good” respectively.

Disagreements on whether SEQ was non-diagnostic were resolved by a referee, a third vascular interventional radiologist. The resolved rating was the median of the three ratings.

### Radiation dose estimation

The dose length product (DLP) and volume CT dose index (CTDIvol) was recorded for each patient. The effective dose (ED) was calculated as follows: ED = DLP*k, using the conversion coefficient k = 0,0056 mSv/mGycm [19].

### Sample size and statistical analysis

Sample size calculation, with a 0.05 level of significance (α) and power (β) 80%, assuming a mean difference of 0.5 points in SEQ with an SD of 1.25, resulted in a sample size of 98 in each group.

Data analysis was performed with Statistical Package for the Social Sciences version 28.0 (IBM) and R version 4.2.0 (The R Foundation).

Differences between experimental and control groups in patient demographics and scan characteristics were assessed by independent samples t-test, and sex by chi-squared test.

Comparisons of OEQ and SEQ were performed with the Wilcoxon rank sum test for unpaired data between the control group and each of the VMIs in the experimental group, and Wilcoxon signed rank test for paired data within the experimental group between VMI at 40 and 50 keV.

Cohen’s D was used as effect size on the log-transformed OEQ data since attenuation, CNR and SNR showed normal distribution and noise was gamma distributed on the log scale. For SEQ the rank-biserial coefficient was reported as effect size. *p* values were inflated by a factor of three to account for the multiplicity of testing. All tests were two-sided, and with a 0.05 level of significance.

The influence of anatomical level, age, sex, and RCT groups on OEQ and SEQ were investigated by multivariable generalized mixed models. Patient ID was included as a random effect allowing for different latent mean values per patient. The log-transformed VA, CNR, and SNR were modelled by Gaussian likelihoods, while the log-transformed noise was modelled by a gamma likelihood, and an ordinal likelihood was used to model the resolved median subjective rating. Reported effect sizes and standard errors are those of the regression coefficients, which are in units of the (log-transformed) response variable per the units of the covariate.

## Results

**Fig. 2:**
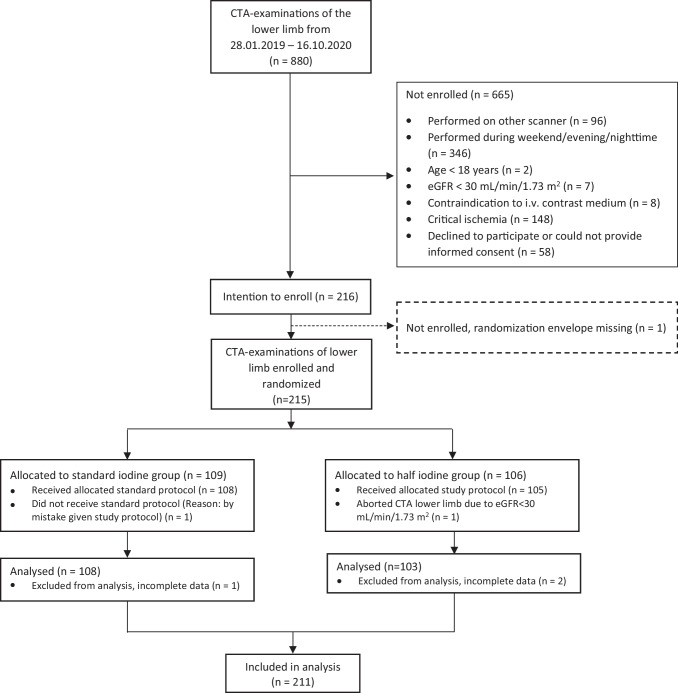
Flowchart of enrolment and randomization

Between 28 January 2019 and 16 October 2020 880, lower limb CTAs were performed. We intended to enrol 216 examinations. However, one randomisation envelope went missing (experimental), and 215 were enrolled. Figure 2 shows a flowchart of enrolment, exclusion, and randomisation. One examination randomized to control received the experimental examination by mistake but was analyzed by intention to treat, according to randomisation. There were four post-randomisation exclusions. One patient was excluded due to eGFR < 30 mL/min/1.73 m^2^. Two examinations were excluded because the spectral base image data were not stored. One examination was excluded because the acquisition only included the abdomen and pelvis and not the lower limbs. During the inclusion period, 13 patients were referred, enrolled, and randomized twice, and one patient three times. Hence, 211 examinations of 182 patients were included in the analysis.

Table 1 shows patient- and scan characteristics in the experimental and control groups.

**Table 1:** Patient and scan characteristics in experimental and control groups: Values are mean (SD), unless otherwise specified

	Experimental group (*n* = 103)	Control group (*n* = 108)	*p* value
Age (years)	70.8 (10.1)	69.5 (11.1)	.39
Sex (proportion male/female)	65/38	66/42	.85
Weight (kg)	79.6 (18.8)	78.8 (19.6)	.78
Contrast volume (mL)	55.4 (13.6)	104.2 (23.3)	< .0001
CM injection rate (mL/s)	5.23 (.82)	5.21 (.93)	.90
Injection time (s)	20.4 (1.29)	20.5 (1.67)	.92
CTDIvol (mGy)	13.4 (13.3)	11.8 (1.69)	.20
DLP (mGy cm)	1711.4 (243.2)	1653.0 (289.8)	.12
ED (mSv)^a^	9.58 (1.36)	9.26 (1.62)	.12

^a^Conversion factor for peripheral CTA = 0.0056 mSv/mGycm

*CM* contrast medium, *CTA* computed tomography angiography, *CTDIvol* CT dose index of volume, *DLP* dose length product, *ED* effective dose

### Primary outcome

Table 2 shows effect sizes and *p* values of differences in VA, CNR, SNR, and noise between experimental and control.

**Table 2:** Effect sizes and *p* values of differences in OEQ (vascular attenuation, image noise, CNR, and SNR) and SEQ between experimental group with VMI at 40 keV and 50 keV and the control group

	Vascular attenuation		Image noise		CNR		SNR		SEQ	
	Effect size^a^	*p*-value	Effect size^a^	*p*-value	Effect size^a^	*p* value	Effect size^a^	*p* value	Effect size^b^	*p* value
Control vs. experimental VMI at 40 keV^c^	1.02	< .0001	.22	.0033	.37	< .0001	.33	< .0001	.11	< .0001
Control vs. experimental VMI at 50 keV^c^	-.057	.022	− .20	.00022	.15	.0058	.16	.0023	.09	< .0001
Experimental VMI at 50 keV vs. Experimental VMI at 40 keV^d^	1.75^e^	< .0001	.90^e^	< .0001	.20^e^	< .0001	.34^e^	< .0001	.80	< .0001

^a^The effect sizes are Cohen’s D on the log-transformed data for OEQ

^b^Biserial rank correlation was used as the effect size for SEQ

^c^*p* values are derived from Wilcoxon’s rank sum test

^d^*p* values are derived from a paired test (Wilcoxon’s signed rank test) and are not directly comparable with the unpaired test between experimental and control

^e^Effect size is paired with Cohen’s D on log-transformed data

*CNR* contrast to noise ratio, *OEQ* objective examination quality, *keV* kiloelectron volt, *SEQ* subjective examination quality, *SNR* signal to noise ratio, *VMI* virtual monoenergetic images

VA, the primary outcome, was higher in the experimental group at 40 keV than in the control group (*p* < 0.0001). VA was higher in the control group compared to the experimental group at 50 keV (*p* = 0.022).

Descriptive statistics showed that VA was consistently higher in the experimental group at 40 keV than in the control group, and experimental at 50 keV across all anatomical levels (Table 3). From the common iliac arteries and further distally the attenuation decreased.

**Table 3:** Descriptive statistics for vascular attenuation, image noise, CNR, and SNR by RCT groups (experimental with VMI at 40 and 50 keV and control) from the abdominal aorta, common iliac-, superficial femoral- and popliteal arteries. Numbers are Median (IQR), unless otherwise specified

			Vascular attenuation			Image noise			CNR			SNR		
RCT group			Control	Experimental		Control	Experimental		Control	Experimental		Control	Experimental	
			120 kVp	50 keV	40 keV	120 kVp	50 keV	40 keV	120 kVp	50 keV	40 keV	120 kVp	50 keV	40 keV
Anatomical Level	AA		430.0 (148.5)	432.0 (163.0)	650.0 (251.5)	29.0 (14.5)	23.0 (12.0)	24.0 (12.5)	12.4 (7.1)	15.9 (8.8)	22.4 (11.0)	14.0 (7.5)	18.2 (8.9)	24.7 (11.7)
	CIA	Left	442.0 (133.0)	418.0 (178.0)	625.0 (251.0)	27.0 (16.0)	23.0 (14.0)	26.0 (17.0)	13.7 (7.5)	15.4 (9.6)	21.3 (15.6)	15.9 (8.5)	18.4 (11.4)	24.3 (17.8)
		Right	444.0 (132.0)	420.5 (180.0)	633.5 (276.3)	26.0 (14.0)	23.5 (14.8)	27.5 (17.8)	12.9 (7.6)	15.2 (9.3)	21.6 (15.3)	14.9 (8.3)	18.6 (10.5)	24.4 (16.1)
	SFA	Left	444.0 (192.8)	403.0 (153.8)	596.5 (249.0)	29.00 (28.5)	30.00 (21.0)	40.50 (37.0)	11.1 (9.1)	12.6 (10.6)	14.7 (12.3)	12.7 (10.5)	14.6 (10.5)	15.6 (13.5)
		Right	453.0 (159.0)	395.0 (127.5)	588.0 (237.5)	31.00 (24.5)	35.00 (39.5)	50.00 (67.0)	11.4 (8.4)	9.2 (8.4)	10.4 (10.3)	13.3 (9.3)	10.9 (9.2)	11.7 (12.8)
	PA	Left	397.0 (160.5)	364.0 (131.8)	536.5 (212.8)	41.0 (50.3)	40.0 (31.3)	60.0 (52. 0)	6.9 (7.8)	7.3 (6.7)	7.7 (7.6)	8.0 (8.9)	8.9 (7.6)	8.7 (8.4)
		Right	401.0 (150.0)	374.0 (139.0)	558.0 (224.0)	47.0 (39.0)	35.0 (39.0)	54.0 (67.0)	7.1 (6.3)	8.8 (9.6)	8.2 (10.6)	8.1 (6.9)	11.1 (12.0)	9.0 (13.1)
Total		^a^	430.5 (155.0)	398.0 (146.5)	596.0 (231.3)	32 (24.0)	28 (23.0)	34 (39.25)	11.2 (8.8)	12.3 (11.0)	15.1 (16.2)	12.8 (9.9)	14.4 (12.7)	16.7 (18.2)
		^b^	5.98 (.33)	5.96 (.36)	6.35 (.40)	3.49 (.57)	2.37 (.62)	3.63 (.73)	2.3 (.62)	2.4 (.69)	2.6 (.84)	2.5 (.59)	2.6 (.68)	2.7 (.80)

^a^Across all anatomical locations and both left and right sides

^b^Numbers are mean (sd) after log-transformation

*AA* abdominal aorta, *CIA* common iliac artery, *CNR* contrast to noise ratio, *IQR* interquartile range, *keV* kiloelectron volt, *kVp* kilovolt peak, *PA* popliteal artery, *RCT* randomized control trial, *SFA* superficial femoral artery, *SNR* signal to noise ratio

**Fig. 3:**
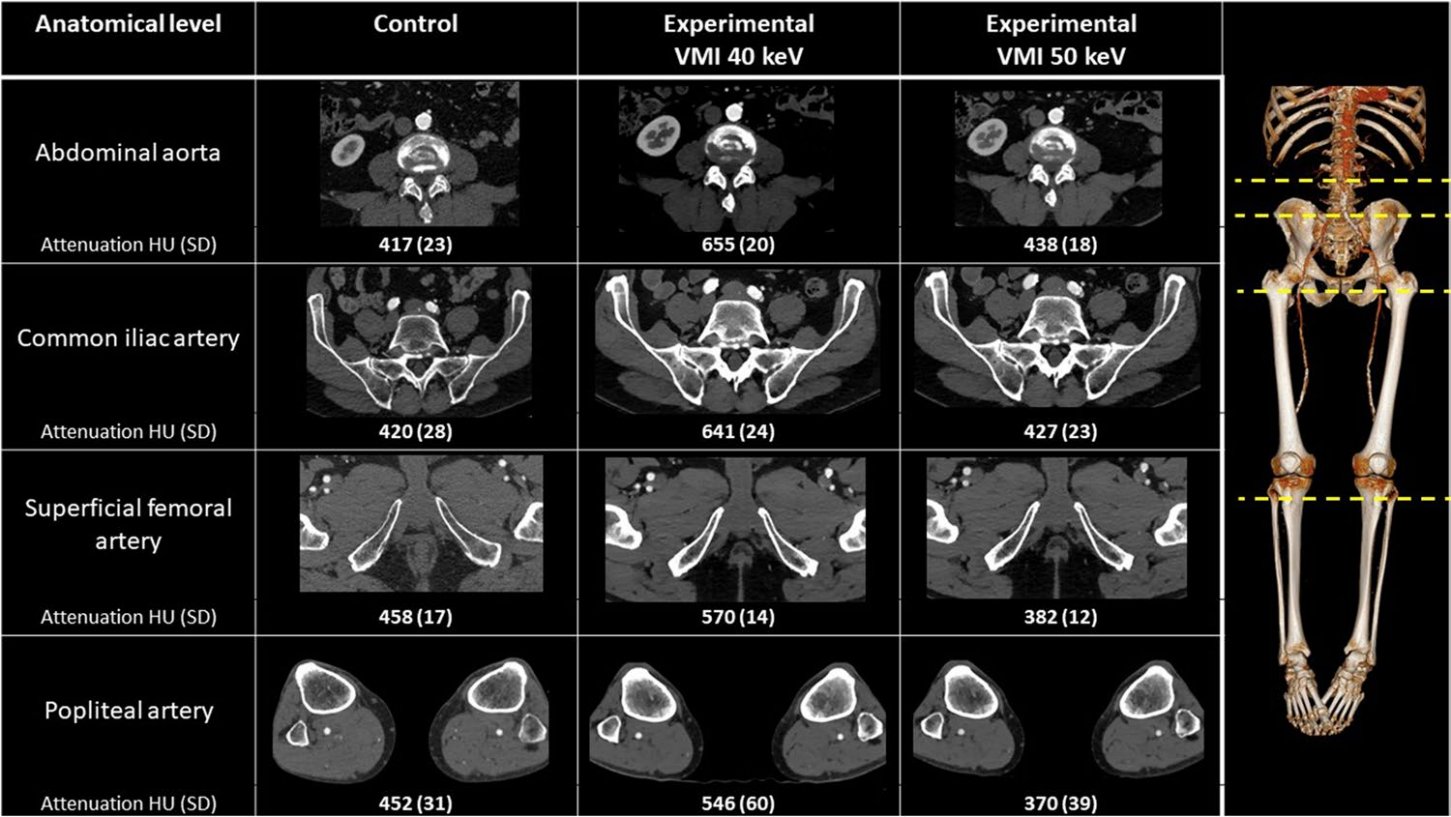
Lower extremity CTA in the control group and in the experimental group with VMI at 50 and 40 keV. Axial images of a 76-year-old male who was referred to lower limb CTA on two occasions and examined with experimental and control protocols. ROIs placed in the abdominal aorta, common iliac artery, superficial femoral artery, and popliteal artery show attenuation and noise values, on conventional 120 kVp (control) and on VMI at 50 and 40 keV (experimental)

Table 4 shows generalized linear models predicting OEQ and SEQ using RCT group and patient factors. The effect of the experimental group remained clear on VMI at 40 keV (*p* < 0.0001), but not on VMI at 50 keV (*p* = 0.44) compared to the control. Figure 3 shows experimental and control examinations performed on the same patient.

**Table 4:** Associations between objective examination quality (vascular attenuation, CNR, SNR, and image noise) and averaged subjective examination quality and patient/examination factors (anatomical level, sex, age, and RCT group)

		CIA^a^	SFA^a^	PA^a^	CA^a^	Sex	Age	Experimental 40 keV^b^	Experimental50 keV^b^
Vascular attenuation	e.s	.0016	− .056	− .15	N/A	.089	.038	.36	− .030
	s.e	.016	.016	.016	N/A	.039	.0019	.038	.0064
	p	.92	.00061	< .0001	N/A	.024	.046	< .0001	.44
Image noise	e.s	.115	.020	− .029	N/A	.0065	− .010	.038	− .034
	s.e	.0075	.0070	.0064	N/A	.012	.0056	.012	.012
	p	< .0001	.0040	< .0001	N/A	.580	.086	.0017	.0047
CNR	e.s	− .54	− .14	.094	N/A	.106	.082	.26	.087
	s.e	.030	.028	.026	N/A	.044	.021	.045	.045
	p	< .0001	< .0001	.00027	N/A	.016	.00012	< .0001	.057
SNR	e.s	− .53	− .13	.10	N/A	.078	.072	.22	.087
	s.e	.029	.027	.025	N/A	.041	.020	.043	.043
	p	< .0001	< .0001	< .0001	N/A	.058	.0004	< .0001	.042
SEQ	e.s	2.55	1.51	.69	.19	− .67	.36	− .69	− .48
	s.e	.17	.15	.14	.17	.52	.25	.51	.51
	p	< .0001	< .0001	< .0001	< .0001	.20	.16	.17	.34

Effect sizes (e.s.), standard errors (s.e.), and *p* values (p) are all derived from the corresponding regression coefficients

^a^effects are compared to the abdominal aorta

^b^effects are compared to the control group (120 kVp)

*CA* calf arteries, *CIA* common iliac arteries, *CNR* contrast to noise ratio, *keV* kiloelectron volt, *kVp* kilovolt peak, *PA* popliteal arteries, *SEQ* subjective examination quality, *SFA* superficial femoral arteries, *SNR* signal to noise ratio

### Secondary outcomes

#### Objective examination quality

Noise was lower in the experimental group at 50 keV than at 40 keV (*p* < 0.0001) and control (*p* = 0.00022), and higher at 40 keV than control (*p* = 0.0033) (Table 2). These effects remained significant in the multivariate analysis (Table 4).

At 40 keV, the experimental group showed higher CNR and SNR than at 50 keV (*p* < 0.0001) and control (*p* < 0.0001). The control showed lower CNR (*p* = 0.0058) and SNR (*p* = 0.0023) than the experimental at 50 keV (Table 2). In the multivariate analysis, only the effects on CNR and SNR between 40 keV and control remained clear (both *p* < 0.001) (Table 4). Table 3 shows OEQ for control and experimental across anatomical levels.

#### Subjective examination quality

**Fig. 4:**
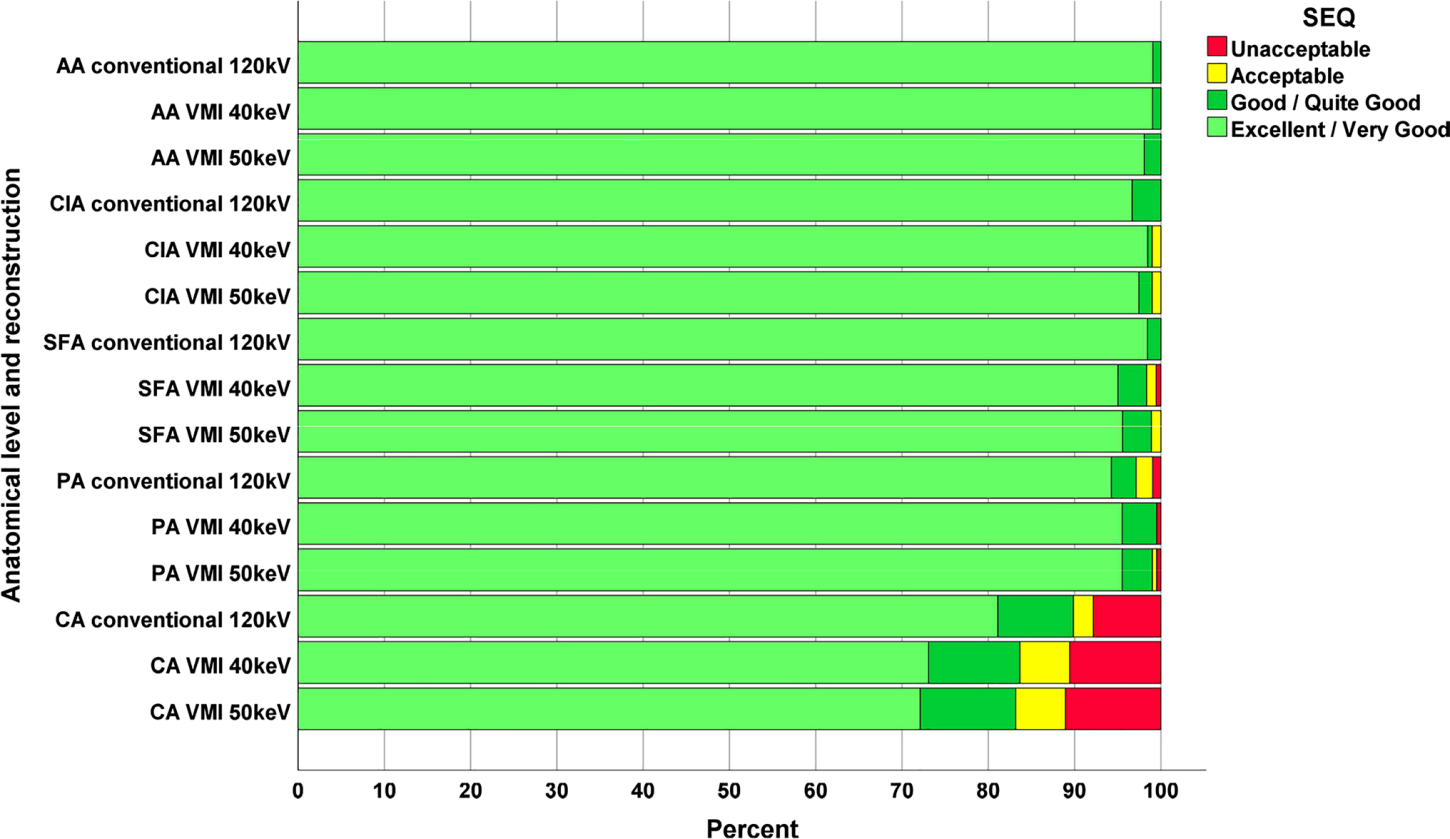
Distribution of subjective examination quality ratings *AA* abdominal aorta, *CIA* common iliac artery, *SFA* superficial femoral artery, *PA* popliteal artery, *CA* calf arteries, *VMI* virtual monoenergetic images, *SEQ* subjective examination quality

The experimental group showed better SEQ at both 40 and 50 keV than the control (*p* < 0.0001) (Table 2). Within the experimental group, 40 keV showed better ratings than 50 keV (*p* < 0.0001). The difference in SEQ between the RCT groups was not significant in the multivariate analysis with an ordinal likelihood (Table 4). SEQ was most frequently rated unacceptable in the calf arteries, in 7.8%, 10.6%, and 11.1% in control, 40 keV and 50 keV, respectively (Fig. 4 and Table 5).

**Table 5:** Distribution of subjective examination quality ratings across anatomical levels and RCT groups

	Anatomical level		Abdominal aorta			Common iliac artery			Superficial femoral artery			Popliteal artery			Calf arteries		
	RCT group		Control	Experimental		Control	Experimental		Control	Experimental		Control	Experimental		Control	Experimental	
	Reconstruction		120 kVp	40 keV^a^	50 keV^a^	120 kVp	40 keV^a^	50 keV^a^	120 kVp	40 keV^a^	50 keV^a^	120 kVp	40 keV^a^	50 keV^a^	120 kVp	40 keV^a^	50 keV^a^
SEQ rating	1 and 1.5	Excellent and very good	99.1%	99.0%	98,1%	96.7%	98.5%	97.4%	98.5%	95.0%	95.6%	94.3%	95.5%	95.5%	81.1%	73.1%	72.1%
	2 and 2.5	Good and quite good	0.9%	1.0%	1.9%	3.3%	0.5%	1.5%	1.5%	3.3%	3.3%	2.9%	4.0%	3.5%	8.8%	10.6%	11.1%
	3	Acceptable	0.0%	0.0%	0.0%	0.0%	1.0%	1.0%	0.0%	1.1%	1.1%	1.9%	0.0%	0.5%	2.3%	5.8%	5.8%
	4	Unacceptable	0.0%	0.0%	0.0%	0.0%	0.0%	0.0%	0.0%	0.6%	0.0%	1.0%	0.5%	0.5%	7.8%	10.6%	11.1%

^a^Virtual monoenergetic images

*keV* kiloelectron volt, *kVp* kilovolt peak, *RCT* randomized controlled trial, *SEQ* subjective examination quality

## Discussion

In this study half iodine load SDCT showed higher VA and noise at 40 keV and lower VA and noise at 50 keV compared to the control, while CNR and SNR were higher at 40 keV.

### Attenuation

We have shown that lower limb CTA with half iodine-load SDCT at 40 keV yielded higher VA across all anatomical levels than full iodine-load conventional CTA. The control showed higher VA than 50 keV, but the effect size was small and the p-value borderline significant, and the difference was not significant in the multivariate analysis. Reported optimal VA is approximately 350 HU, while 200–250 HU is clinically acceptable [20, 21]. In our study, the mean VA exceeded 350 HU in both RCT groups, at both VMIs and at all anatomical levels.

### Image noise

Noise was lower at 50 keV than in the control, but the effect size was small. Noise was higher at 40 keV than both 50 keV and control with large and small effect sizes, respectively. This difference in effect size is partially due to the paired comparison between 40 and 50 keV. Thus, with different VMIs, DECT allows for both higher attenuation and lower noise, since both VMIs are available to the radiologist in clinical practice,

### CNR and SNR

CNR and SNR were higher at 40 keV than at 50 keV and control with very small *p* values and small-to-medium effect sizes. The control showed the lowest CNR and SNR, and 50 keV was in between, with small *p* values and small-to-medium effect sizes. This indicates that the increase in attenuation at lower keV-values is larger than the increase in noise.

### Subjective examination quality

SEQ was best at 40 keV with a small effect size compared to the control group and a large effect size compared to 50 keV, again partially due to paired testing. This might indicate that noise is less important to SEQ than attenuation, although this effect may be specific to CTA [22].

More examinations were rated as 3 (acceptable) or 4 (unacceptable) in the calf arteries than other anatomical levels. In the calf arteries, 7.8% were rated unacceptable in the control group compared to 10.6% and 11.1% in the intervention group at 40 and 50 keV, respectively. This might be due to blooming artefacts at low VMIs in calcified arteries. We did not test this difference statistically, but it may well be clinically significant as the calf and foot are often the areas of interest. The lower performance in distal vessels will have to be addressed by further protocol optimization.

Unfortunately, we cannot compare SEQ findings from the calf with objective measurements. We decided against measuring in the calf because it would be difficult to adequately place ROIs in very small and calcified vessels. Although some studies have made the same decision, others have made measurements of arterial attenuation in both calf and foot [23].

### Acquisition techniques

Previous single energy CTA studies with 2/3^rd^ CM dose have achieved higher attenuation, SNR, and CNR at 70 kVp but lower at 80 kVp, compared to full CM dose at 120 kVp, but no significant difference in subjective examination quality [24, 25].

A smaller study by Ren et al, which used a similar technique to ours, concluded that half of CM DECT yielded higher attenuation, CNR and SNR and lower noise at both 40 and 50 keV than standard CM at 120 kVp [26]. However, that study included only 40 patients and did not have sufficient statistical power to show significant differences neither in attenuation at 40 or 50 keV, nor noise at 40 keV. We included 211 examinations and were able to show significantly improved attenuation at 40 keV and significantly reduced noise at 50 keV.

### Contrast administration techniques

CM administration technique is crucial for examination quality in CTA, and there are large variations in protocols. Ren et al used a fixed CM volume of 90 or 45 mL, while Almutairi et al used a weight-tailored CM dosage of 1.5 or 0.75 ml/kg [26, 27]. Both used 350 mgI/ml CM and their mean CM doses were similar to ours. In this study, we used a weight-tailored CM dosage. Individually tailored CM dosage may reduce iodine load and variation in enhancement compared to fixed dosage [28–30], and total body weight is the most commonly used strategy [31].

We replaced the reduced contrast volume in the experimental group with saline, keeping the injected volume, injection rate, CM concentration, and saline chaser unchanged, thus reducing the room for errors. Since we had no weight-based exclusion criteria, our results should be valid for the target population [30].

### Clinical implications

Regular optimization of CT protocols is crucial to adapt CM injection protocols to evolving scanner technology, which requires less CM, without compromising examination quality. Although our study focused on lower limb CTA, our results might apply to other vascular CT examinations. We have shown that it is feasible in everyday clinical practice to halve the iodine load with SDCT CTA and sustain or even improve examination quality compared to 120 kVp conventional CT. This may allow us to reduce the risk of PC-AKI, examine patients with more severe kidney impairment and provide higher quality examinations or salvage poor examinations when impaired kidney function limits the CM dose [18].

In response to the ongoing global shortage of iodinated contrast media, DECT is one of the suggested approaches to reduce CM consumption. Although the number of DECT scanners are limited, and many facilities do not have access to DECT scanners, this might be expected to improve in the time to come.

### Limitations

Our study was performed at a single centre and on one scanner only, limiting the external validity of our results for other DECT scanners. We limited our study to VMI reconstructions at 40 and 50 keV since previous studies identified these as the most promising [18, 26]. Including VMI at smaller intervals might have identified an optimal VMI between 40 and 50 keV. Since the experimental group showed less attenuation on conventional images, it was impossible to achieve complete blinding of the personnel assessing OEQ or SEQ to the RCT groups. Our measurements may have been influenced by clinical factors such as cardiovascular state, stenoses, or occlusions, but such influence is expected to be minimized by randomization. Although four patients were excluded after randomisation, we believe the risk of bias is small considering that the reasons for exclusions were not related to the outcomes, the large number of participants, and the strength of the observed effects.

### Conclusion

Half iodine load SDCT lower limb CTA at 40 keV achieved higher vascular attenuation compared to conventional 120 kVp with standard iodine-load. CNR, SNR, noise, and subjective examination quality were higher at 40 keV while 50 keV showed lower noise levels.

## Abbreviations

AA: Abdominal aorta
CIA: Common iliac arteries
CM: Contrast medium
CNR: Contrast-to-noise-ratio
CTA: Computed tomography angiography
CTDIvol: Volume CT dose index
DECT: Dual-energy CT
DLP: Dose length product
ED: Effective dose
eGFR: Estimated glomerular filtration rate
HU: Hounsfield units
keV: Kiloelectron volt
kVp: Kilovolt peak
Noise: Image noise
OEQ: Objective examination quality
PA: Popliteal artery
PAD: Peripheral artery disease
PC-AKI: Post-contrast acute kidney injury
RCT: Randomized controlled trial
ROI: Region of interest
SDCT: Dual-layer spectral detector CT
SD: Standard deviation
SEQ: Subjective examination quality
SFA: Superficial femoral arteries
SNR: Signal-to-noise-ratio
VA: Vascular attenuation
VMI: Virtual monoenergetic images

## Declarations

### Guarantor

The scientific guarantor of this publication is Peter Mæhre Lauritzen.

### Statistics and biometry

One of the authors has significant statistical expertise.

### Methodology

• prospective

• randomized controlled trial

• performed at one institution
